# Hemophilia in Mexico: Updated Consensus Recommendations on Diagnosis, Treatment and Gene Therapy

**DOI:** 10.3390/diseases14070259

**Published:** 2026-07-17

**Authors:** Martha Alvarado Ibarra, Alma B. Mera-González, Ana L. Tapia-Enriquez, Ana P. Ramirez-Hoyos, Annel Martínez-Ríos, Atenas Villela-Peña, Carlos Martínez-Murillo, Carolina F. Cruz-García, Carolina García-Castillo, Cristina E. Madera-Maldonado, Daniel Cabello-Modesto, David Ávila-Castro, Eleazar Hernández-Ruiz, Emmanuel R. Rodríguez-Cedeño, Eugenia P. Paredes-Lozano, Faustino Leyto-Cruz, Fernando Montero-Palomo, Fernando Perez-Zincer, Flavio Rojas-Castillejos, Geraldin M. Gutiérrez-Gómez, Gonzalo Iván Gómez López, Irene Anaya-Cuellar, Israel Cervantes-Sánchez, Jaime García-Chávez, Javier de Jesús Morales Adrián, J. Antonio De la Peña-Celaya, José L. Alvarez-Vera, José L. López-Arroyo, Josué I. Ruiz-Contreras, Juan M. Pérez Zúñiga, Juan P. Macías Flores, Karina Silva-Vera, Laura E. Merino Pasaye, Leire Montoya Jiménez, Luara L. Arana-Luna, Lucy González-Villarroel, M. Cecilia Gómez-Núñez de Cáceres, Maria D. Valencia Rivas, M. Eugenia Espitia-Ríos, M. Raquel Miranda-Madrazo, Nishalle Ramírez-Muñiz, Óscar Teomitzi-Sánchez, Pablo A. García Chávez, Ramón A. Bates-Martín, Roberto Ovilla Martínez, Sergio J. Loera-Fragoso, Yessica Torres-Giron, Alberto Villalobos-Prieto, Lénica A. Chávez-Aguilar, W. Herrera-Olivares

**Affiliations:** 1 Hospital Angeles Lomas, Huixquilucan 52763, State of Mexico, Mexico; paulinaramirezhoyos@gmail.com (A.P.R.-H.); cabellomod@gmail.com (D.C.-M.); eriol_db@hotmail.com (E.R.R.-C.); fperezincermx@yahoo.es (F.P.-Z.); ivangomezfm@hotmail.com (G.I.G.L.); nishalle.ramirez@gmail.com (N.R.-M.);; 2 Hospital del Niño DIF Pachuca, Pachuca 42080, Hidalgo, Mexico; almglez36@gmail.com; 3 Hospital General ISSSTE Presidente Lázaro Cárdenas, Chihuahua 31160, Chihuahua, Mexico; laura_tapi@hotmail.com (A.L.T.-E.); irene_ldmf@hotmail.com (I.A.-C.); 4 Hospital Regional No. 1 ISSSTE “General Ignacio Zaragoza”, Mexico City 09220, Mexico; an_mar71@yahoo.com.mx (A.M.-R.); atenas.villela@gmail.com (A.V.-P.); mvrivas83@gmail.com (M.D.V.R.); oscar.teomitzi@hotmail.com (Ó.T.-S.); 5 Hospital General de México Dr. Eduardo Liceaga, Mexico City 06720, Mexico; carlosmtzmurillo@gmail.com (C.M.-M.); pablo.macias00@gmail.com (J.P.M.F.); 6 Hospital Juárez de México, Mexico City 07760, Mexico; carol_260687@hotmail.com; 7 Hospital Central Militar, Mexico City 11200, Mexico; carohematohcm@gmail.com (C.G.-C.); jirc001@gmail.com (J.I.R.-C.); psiquis_80@hotmail.com (M.C.G.-N.d.C.); 8 Hospital de Alta Especialidad de Ixtapaluca, Ixtapaluca 56530, State of Mexico, Mexico; hematofem@gmail.com; 9 Hospital de Alta Especialidad de Veracruz, IMSS Bienestar, Veracruz 91910, Veracruz, Mexico; liquiddac@yahoo.com.mx; 10 Hospital Regional Presidente Juárez, Oaxaca de Juárez, Oaxaca 68024, Oaxaca, Mexico; eleazar_li@hotmail.com; 11 Hospital General de Zona No. 1 IMSS, Aguascalientes 20270, Aguascalientes, Mexico; genios280198@hotmail.com; 12 Hospital Licenciado Adolfo López Mateos, ISSSTE, Mexico City 01030, Mexico; fleyto@yahoo.com.mx; 13 Hospital Militar de Especialidades de la Mujer y Neonatología, Mexico City 11200, Mexico; femopa88@gmail.com (F.M.-P.); ysykatorres43@gmail.com (Y.T.-G.); 14 Centro Médico CESAT, Santo Domingo Tehuantepec 70760, Oaxaca, Mexico; flavius077@gmail.com; 15 Hospital General ISSSTE Tláhuac, Mexico City 13273, Mexico; margarita.gutierrez8910@gmail.com; 16 Hospital General ISSSTE Tlaxcala, San Gabriel Cuauhtla 90117, Tlaxcala, Mexico; iscervantes@hotmail.com; 17 Hospital de Especialidades del Centro Médico Nacional La Raza, Mexico City 02990, Mexico; jaimegch2@gmail.com; 18 Centro Médico Pensiones, Mérida 97070, Yucatán, Mexico; jamoad2@live.com.mx; 19 Centro Médico Nacional 20 de Noviembre, ISSSTE, Mexico City 03104, Mexico; dr.delape.hemato@gmail.com (J.A.D.l.P.-C.); draselo@hotmail.com (J.L.A.-V.); kbzaz@yahoo.com (J.M.P.Z.); leire_1229@hotmail.com (L.M.J.); luuaraa@gmail.com (L.L.A.-L.); merrmeri2@yahoo.com.mx (M.E.E.-R.); racuel1790@yahoo.com (M.R.M.-M.); 20 Hospital General “B”, ISSSTE, Ciudad Juárez, Chihuahua 32315, Chihuahua, Mexico; lpzarroyo@hotmail.com; 21 Hospital General ISSSTE, Tampico 89137, Tamaulipas, Mexico; ksilvav68@gmail.com; 22 Clínica Hospital ISSSTE Xalapa, Xalapa-Enríquez 91020, Veracruz, Mexico; sketch0712@gmail.com; 23 Hospital Regional 1° de Octubre, ISSSTE, Mexico City 07760, Mexico; gonzalezvillarroellucy@gmail.com (L.G.-V.); nomarsetab@gmail.com (R.A.B.-M.); 24 Hospital Regional General 02 IMSS, Ciudad Juárez 32424, Chihuahua, Mexico; pablogarcia1720@gmail.com; 25 IMSS Hospital General Regional 25, Mexico City 09100, Mexico; 26 Hospital Dr. Santiago Ramón y Cajal, ISSSTE, Durango 34070, Durango, Mexico; sloera51@yahoo.com.mx; 27 Centro de Cancer Centro Médico ABC, Observatorio, Mexico City 01120, Mexico; avillalobosabc@yahoo.com.mx; 28 Hospital Regional de Alta Especialidad Puebla, Puebla 72570, Puebla, Mexico; lenicachavez@gmail.com (L.A.C.-A.); wheerol@hormail.com (W.H.-O.)

**Keywords:** hemophilia A, hemophilia B, prophylaxis, inhibitors, gene therapy, Delphi method, coagulation factor concentrates, non-factor therapies, Mexico

## Abstract

Hemophilia is an X-linked inherited bleeding disorder, classified as type A or type B. Therapeutic advances offer new treatment options that improve disease control and reduce associated complications, including inhibitor development and hemophilic arthropathy. This document aims to update the Mexican hemophilia consensus, reviewing current evidence on diagnosis and management, and addressing gaps in the treatment and follow-up of patients in Mexico, aligning local needs with international recommendations. A PubMed literature search covering the last five years (up to September 2025) was conducted, prioritizing consensus statements, guidelines, and systematic reviews. Using the Delphi methodology, a structured questionnaire was submitted electronically to forty-four experts. Aspects without initial agreement were discussed at an in-person meeting. Consensus was defined as at least 80% of votes in favor. Recommendations were issued across six domains: laboratory diagnosis, genetic testing, management of hemophilia A and B without and with inhibitors, adjuvant treatments, and gene therapy. The recommendations address prophylaxis with coagulation factor concentrates, non-factor therapies, immune tolerance induction, perioperative management, pain management, and eligibility criteria and follow-up protocols for gene therapy with adeno-associated viral vectors. This consensus provides updated, evidence-based recommendations adapted to the Mexican healthcare context, identifying priority areas, including timely access to non-factor therapies and gene therapy, development of a national referral network for complex cases, and inclusion of novel therapeutic agents in the institutional essential medicines list, with the aim of improving the quality of life of people with hemophilia in Mexico.

## 1. Introduction

Hemophilia is a rare hereditary X-linked bleeding disorder characterized by a partial or complete deficiency of coagulation factor (F)VIII in hemophilia A or FIX in hemophilia B caused by pathogenic variants affecting the corresponding genes [1]. The current cornerstone of treatment for people with hemophilia (PwH) is intravenous (IV) supplementation of the deficient factor, administered either on demand or prophylactically, with the main objective of preventing spontaneous hemarthroses and other forms of bleeding, including muscular, mucosal, gastrointestinal and intracranial hemorrhages, although this carries a considerable risk of inhibitor development [2]. In recent years, new therapies have emerged, such as rebalancing therapies or mimetic antibodies, with mechanisms of action different from that of replacement therapy and high rates of zero bleeding, offering new treatment perspectives for the benefit of patients (Table 1).

However, the lack of infrastructure and limited resources constitute significant barriers to their use in Mexico.

The objective of this document is to update the Mexican hemophilia consensus published in 2021, focusing on the best available therapies and/or phase 3 data available to date, and to provide the relevant recommendations to help ensure that patients receive appropriate and timely care, avoiding, as far as possible, hemorrhagic complications that may affect patients’ survival and quality of life.

## 2. Methodology

For the development of this consensus, a literature search was conducted in PubMed covering the last five years (up to September 2025) in English and Spanish, giving priority to consensus statements, guidelines, literature reviews and/or systematic reviews. Using the Delphi method, a questionnaire was prepared and sent electronically to the forty-four experts participating in the consensus. The validation and compilation of the questionnaire responses were consolidated by the coordinator.

Based on the responses from the panel of experts, a manuscript was drafted for review by the coordinator. Aspects for which no initial agreement was reached were discussed at an in-person meeting and resolved by consensus. Consensus was defined as obtaining at least 80% of votes in favor. Once consensus had been reached, the manuscript was updated for review and final validation by the project coordination.

## 3. Diagnosis

### 3.1. Laboratory Tests

Recommendation 1. In screening studies, when hemophilia is suspected, the complete blood count should be within the reference parameters if no other justifiable abnormality exists (with 95.45% agreement).

The prothrombin time (PT) should have a value not exceeding 2.4 s in relation to the control, and the activated partial thromboplastin time (aPTT) should have a value greater than eight seconds in relation to the control (with 100% agreement). Mexican studies have shown that shortening the aPTT to 3 to 5 s improves the specificity of the test, provided that the clinical presentation is suggestive of a bleeding disorder [3].

Recommendation 2. In congenital hemophilia, the mixing test of patient plasma with pooled plasma in a 1:1 ratio should correct the aPTT value to normal ranges. Failure to correct indicates the presence of an anticoagulant inhibitor in the patient’s plasma (with 97.73% agreement).

Recommendation 3. In patients with no hereditary history of hemophilia, presenting with a hemorrhagic clinical picture and prolonged aPTT, correction studies with plasma should be performed (confirming a pattern of factor deficiency) together with confirmatory studies of coagulation factor (CF) activity using a coagulometric or chromogenic assay for FVIII and a coagulometric assay for FIX (with 100% agreement).

Recommendation 4. In newborns with a family history of hemophilia, confirmatory studies for quantification of FVIII and FIX should involve targeted measurement of the relevant coagulation factor in either umbilical cord blood or peripheral blood samples obtained from the neonate (with 93.18% agreement).

Recommendation 5. In patients previously treated with coagulation factor concentrates (CFC), testing for inhibitors should be performed in the event of changes in the bleeding phenotype and prior to scheduled surgical procedures (with 97.73% agreement).

Without reaching the established agreement threshold of 80% to issue a recommendation, 70% of the expert panel agreed on screening for inhibitors using the Bethesda–Nijmegen technique in PwH without prior exposure to CFC at 20, 50, 100 and 150 exposure days (ED). Considering that 79% of inhibitor antibodies occur within the first 20 ED and the remaining 21% within 75 ED [4], each hemophilia treatment center will establish inhibitor monitoring according to the accessibility of the test.

#### Discussion

Hemostatic testing plays a key role in both the diagnosis and clinical monitoring of PwH; therefore, access to these laboratory tests is essential for the management of these patients, including the determination of inhibitors. Traditional one-stage coagulation assays used to measure FVIII and FIX levels remain fundamental, although they present diagnostic limitations in certain cases and difficulties in monitoring new therapies. Recent consensus statements recommend combining one-stage assays with chromogenic assays (or two-stage assays) to achieve more accurate measurement, depending on the CFC used [2,5].

The clinical presentation of patients who have developed inhibitors includes a higher frequency of bleeding in those receiving prophylactic therapy with CFC, refractory bleeding despite administration of CFC, or a lower-than-expected recovery of factor levels following infusion of CFC. Initial laboratory suspicion may also include the finding of a prolonged aPTT without an apparent cause, which is inhibited in mixing studies with normal plasma [6].

Assays to detect the presence of inhibitors are recommended, particularly in patients with lack of response to supplementation, mainly from a clinical perspective. Since the presence of inhibitors may occur within the first 20–30 days after initiation of CFC, it may be advisable to perform aPTT after factor infusion and subsequently carry out inhibitor characterization [6]. In children, the World Federation of Hemophilia (WFH) recommends monitoring for inhibitors until reaching 20 days post-exposure, every 10 exposure days until reaching 50 exposure days, and at least twice per year until reaching 150 exposure days [7].

### 3.2. Genetic Study

Recommendation 1. Centers with available diagnostic resources should perform genetic profiling of PwH, beginning with testing for inversion 1 and inversion 22 in the case of hemophilia A; if negative, complete sequencing of the FVIII gene should be performed (with 95.45% agreement).

Recommendation 2. For hemophilia B, sequencing of the FIX gene should be performed to detect mutations or deletions (with 95.24% agreement).

#### Discussion

Genetic assessment in hemophilia is essential for understanding disease pathophysiology, confirming the diagnosis in complex cases, predicting the likelihood of inhibitor formation, identifying female carriers and offering prenatal diagnosis and genetic counseling when appropriate [4]. Approximately 80% of mothers of sporadic hemophilia cases are carriers of a pathogenic variant, whereas the remaining cases are thought to result from X chromosome mosaicism.

Prenatal diagnosis is an important component of the management of hemophilia carriers and may influence decisions regarding pregnancy continuation. Fetal evaluation can be performed using non-invasive approaches, including sex determination through analysis of cell-free fetal DNA in maternal blood during the first trimester or ultrasonography after 15 weeks of gestation, although these methods are not conclusive. Invasive diagnostic procedures, such as chorionic villus sampling at 10–12 weeks of gestation or amniocentesis between 15 and 18 weeks, may also be used [2].

Genetic testing is recommended in the following cases [4]:

- Patients with a clinical or biochemical diagnosis of hemophilia (to confirm the diagnosis and characterize the genetic variant).
- Male relatives of carrier women (to determine whether they are affected).
- Women with a family history of hemophilia (to determine their carrier status).
- Partners of carriers or of men with hemophilia who are planning a pregnancy.
- Pregnancies in which the fetus is at risk of hemophilia, to facilitate prenatal diagnosis and planning of delivery.
- Genetic analysis should be initiated in an affected individual whenever possible. In hemophilia A, initial genetic screening studies investigate inversion of introns 22 and 1.

If these variants are absent, comprehensive sequencing of the FVIII gene is subsequently carried out. For hemophilia B, mutation analysis generally consists of sequencing the eight exons of the FIX gene to identify pathogenic variants or deletions [8,9]. If no causal variant is identified, other genes may be analyzed or non-classical mechanisms considered (e.g., somatic mosaicism).

The use of next-generation sequencing (NGS) as a first-line method is recommended, as it allows analysis of entire genes (including introns and regulatory regions), although access to this method is limited in developing countries such as Mexico. Complementary techniques may also be used, such as multiplex ligation-dependent probe amplification (MLPA) to detect large rearrangements. Variants should be classified according to international criteria (e.g., ACMG/AMP): pathogenic, likely pathogenic, of uncertain significance, likely benign, or benign. It is essential to correlate genetic findings with factor levels and the clinical history [4].

As inherited disorders, hemophilia A and hemophilia B have significant medical, psychological and emotional consequences for both affected males and female carriers. The risk of passing the condition to offspring may lead to feelings of guilt among parents and can strongly affect reproductive decision-making. Genetic counseling provides patients and their families with information regarding the inheritance patterns of these disorders and supports informed medical and personal choices. Consequently, it is considered an essential component of comprehensive hemophilia care [9].

## 4. Treatment

Recommended management of hemophilia consists of intravenous replacement therapy with FVIII for hemophilia A and FIX for hemophilia B, administered either prophylactically or on demand. Currently, the application frequency ranges from 5 to 7 days for extended half-life factor VIII to 7 to 14 days in the case of extended half-life factor IX [10,11]. However, several limitations of conventional replacement therapy, including the development of inhibitory antibodies, poor adherence to intravenous administration, challenges related to venous access, geographical barriers to treatment and variable hemostatic control with standard prophylaxis, prompted the development of non-factor therapies requiring less frequent subcutaneous administration (Table 1).

Emicizumab was the first approved non-factor prophylactic treatment for hemophilia A. However, it is not indicated for hemophilia B and has been linked to thromboembolic complications and thrombotic microangiopathy when administered together with a high dose (>100 mg/kg/day) of prothrombin complex concentrate [12]. Similar thromboembolic complications have also been described with other non-factor therapies targeting tissue factor pathway inhibitor (TFPI), such as concizumab and befovacimab.

Concizumab is approved for hemophilia A and B with inhibitors, although three non-fatal thromboembolic events were reported during the phase 3 study in participants receiving concomitant therapy and presenting established risk factors [13]. Befovacimab, which was being investigated for hemophilia A and B, with or without inhibitors, had its clinical development terminated after three cases of thrombosis in the central nervous system occurred during the phase 2 study [14]. Fitusiran, an antithrombin siRNA therapy, has been approved for hemophilia A and B regardless of inhibitor status. The phase 2 study of fitusiran was temporarily halted following a fatal cerebral venous sinus thrombosis and several additional non-fatal thrombotic events observed in other clinical studies within the fitusiran clinical program. These complications were associated with known risk factors, including use of coagulation factor concentrates or bypassing agents, and antithrombin levels < 10%. To reduce thrombotic risk, the dosing protocol was modified by introducing an antithrombin-guided strategy aimed at maintaining antithrombin levels between 15% and 35%, together with recommendations for lower doses of procoagulant agents to manage breakthrough bleeding [15]. With these adjustments, only a limited number of thrombotic events were observed in the phase 3 open-label extension study, including one event considered treatment-related among four of the 213 participants.

Consequently, there is still an unmet need for alternative hemophilia therapies with a more favorable risk–benefit profile. Marstacimab is a recently approved monoclonal antibody in the United States indicated for prophylactic treatment in patients with severe hemophilia A or moderate to severe hemophilia B. Marstacimab targets TFPI, thereby reducing inhibition of activated FX and the tissue factor–FVII complex, which enhances thrombin generation and clot formation independently of FVIII and IX [16].

This efficacy was shown in the phase 3 BASIS study, in which marstacimab reduced the annualized bleeding rate to 5.09 in patients previously on routine factor prophylaxis [16].

### 4.1. Patients with Hemophilia A Without Inhibitors

Recommendation 1. People with severe and moderately severe hemophilia (baseline FVIII level < 1 and between 1 and 5 IU/dL, respectively) should be offered prophylactic management rather than treatment on demand (level of agreement 93.19%), as should people with hemophilia with a severe bleeding phenotype and a baseline FVIII level > 2 IU/dL (with 93.19% agreement).

Recommendation 2. In settings with limited CFC resources where standard-dose prophylaxis is not possible, low-dose FVIII prophylaxis is suggested instead of episodic treatment of bleeding events (with 100% agreement).

Recommendation 3. Emicizumab should be considered as a therapeutic option with a lower treatment burden than CFC, particularly in patients with problems related to venous access and poor adherence to CFC (with 100% agreement). Initiation of treatment with emicizumab after diagnosis should be considered and discussed with parents in all children or adults with severe hemophilia A, unless it is not economically viable (with 86.36% agreement).

Recommendation 4. Extended half-life recombinant FVIII (rFVIII) concentrates offer a lower treatment burden than standard half-life concentrates (with 97.73% agreement).

Recommendation 5. Efanesoctocog alfa, when available and affordable, should be seriously considered instead of standard CFC, given its substantially longer half-life and lower annual bleeding rate (with 97.73% agreement).

Recommendation 6. Marstacimab or concizumab, both anti-TFPI agents, should be considered as therapeutic options, depending on their availability, since their subcutaneous administration, once weekly for marstacimab or every 24 h for concizumab via a prefilled pen, reduces the treatment burden for patients (with 88.64% agreement).

Recommendation 7. People with hemophilia who require surgery should be treated by a multidisciplinary team with appropriate expertise in the disorder (with 100% agreement).

Recommendation 8. All FVIII concentrates are approved for the prevention of bleeding in major surgery and may be used in any surgical context (with 81.82% agreement).

Recommendation 9. In people with hemophilia undergoing a major invasive procedure, continuous infusion or bolus infusion of standard half-life rFVIII concentrates or plasma-derived concentrates is suggested in those without high-responding inhibitors (with 90.90% agreement).

#### Discussion

It is widely accepted that prophylactic use of CFC provides substantial benefit by reducing bleeding events with minimal adverse events. However, the cost and access to the various CFC represent a significant barrier to the implementation of this recommendation [1,2].

In addition to people with severe hemophilia, for whom the recommendation for prophylaxis is undisputed, the publication by Young [17] has suggested that prophylaxis could be indicated in people with hemophilia with baseline FVIII levels between 2 and 5 IU/dL, before the manifestation of a severe bleeding phenotype, or even in those with a baseline FVIII level of 5 to 15 IU/dL and a severe bleeding phenotype. Although there is very little information about patients with mild and moderate hemophilia with joint damage or bleeding phenotype, the literature review by Di Minno et al. found that up to 77% of patients with moderate hemophilia have joint damage and reported joint bleeding [18]. It is because of these groups that it has currently been suggested to raise protection levels above 3–5% [19]. The expert group did not reach consensus for recommending prophylaxis in any of the scenarios presented with FVIII levels between 2 and 5 IU/dL or between 5 and 15 IU/dL. There is evidence that subcutaneous (SC) administration of emicizumab and marstacimab reduces treatment burden and has a significant impact on improved adherence and quality of life, which is essential for the success of prophylaxis. The latest International Society of Thrombosis and Hemostasis (ISTH) guidelines from 2024 warned of the lack of certainty regarding the efficacy and safety of emicizumab in children, but the HAVEN 7 study has recently demonstrated its efficacy and good tolerability in the pediatric population [1,20,21,22].

Similarly, the recommendation for the use of extended half-life CFC reflects their lower treatment burden due to less frequent injections, improving adherence and therefore effective control of bleeding risk [1,23].

In recent years, new therapies have been approved, including efanesoctocog alfa, an ultra-extended half-life FVIII concentrate; modulators of the natural regulators of coagulation (concizumab, marstacimab, fitusiran); and, more recently, gene therapy [17]. It would be advisable to incorporate these new therapies into official formularies in a timely manner, based on the best available scientific evidence. Efanesoctocog alfa demonstrated efficacy in preventing bleeding, near-normal FVIII activity and improvements in physical health, pain and joint health in the phase 3 XTEND-1 study [24].

The phase 3 BASIS study demonstrated the safety and effectiveness of a weekly injection of marstacimab in reducing bleeding events over more than 12 months, with an additional extension period of 16 months. The trial included participants aged 12 to 74 years with severe hemophilia A (FVIII < 1%) or moderate to severe hemophilia B (FIX ≤ 2%), without inhibitors, who were followed during a 6-month observation period under either on-demand prophylaxis or routine prophylaxis and then received 150 mg of marstacimab administered subcutaneously once weekly during a 12-month active treatment phase. Overall, treatment led to a 35% reduction in annualized bleeding rate (ABR) in patients receiving routine factor prophylaxis and a 92% reduction in those managed on demand. In the on-demand group (*n* = 33), the mean ABR decreased from 39.86 (95% CI, 33.05–48.07) during observation to 3.20 (95% CI, 2.10–4.88) during active treatment phase, demonstrating statistical superiority of marstacimab (estimated ratio 0.080 [95% CI, 0.057–0.113]; *p* < 0.0001). In the routine prophylaxis cohort (*n* = 83), the mean ABR decreased from 7.90 (95% CI, 5.14–10.66) to 5.09 (95% CI, 3.40–6.78, meeting both non-inferiority and superiority criteria (estimated difference −2.81 [95% CI, −5.42 to −0.20]; *p* = 0.0349). Regarding safety, no deaths or thromboembolic events were reported, and marstacimab was generally safe and well tolerated throughout the study period [16,25].

The phase 3 explorer8 study supported the approval of concizumab. In this trial, concizumab-mtci prophylaxis reduced ABR by 86% in patients with hemophilia A without inhibitors and by 79% in those with hemophilia B without inhibitors compared with no prophylactic treatment. Following the introduction of risk mitigation strategies, no thromboembolic events were observed [26]. Finally, fitusiran was evaluated in the ATLAS program, which observed a 73% reduction in the annual bleeding rate in patients who had developed antibodies against the factors used as therapy and a 71% reduction in patients who had not developed antibodies, compared with previous on-demand treatment [27,28].

Given the mechanism of action of tissue factor pathway inhibitor inhibitors and the available evidence from phase 2 and phase 3 clinical studies, in which thrombotic events have been observed mainly in association with concomitant risk factors and the simultaneous use of procoagulant agents, it is appropriate to consider targeted monitoring strategies in patients treated with concizumab or marstacimab [16].

It is recommended that all patients eligible for these therapies undergo baseline evaluation and periodic clinical monitoring aimed at identifying symptoms suggestive of thrombosis and systematic assessment of thrombotic risk factors. In asymptomatic patients without additional risk factors, close clinical monitoring may be sufficient.

In patients with thrombotic risk factors, a history of thromboembolic events or a need for concomitant treatment with procoagulant agents, periodic monitoring of basic thrombotic markers is suggested, including D-dimer and fibrinogen, as complementary tools for the early detection of coagulation activation.

In the event of persistent elevation of these markers or the appearance of clinical manifestations suggestive of thrombosis, targeted imaging studies are recommended to rule out subclinical thrombosis and allow timely adjustment of the therapeutic strategy, with the aim of optimizing the benefit–risk profile of anti-TFPI therapies.

Conversely, there is substantial evidence in the literature regarding the perioperative management of major surgery in PwH. Compared with patients without hemophilia, these cases require more extensive planning and closer coordination with the healthcare team. Accordingly, all guidelines recommend that such patients be managed in a comprehensive hemophilia treatment center or in consultation with one [1,4]. This is particularly important with the increasing availability of extended half-life factor concentrates in hospital and emergency care settings, making it vitally important to standardize the doses of these preparations in international guidelines and their use in acute bleeding due to the current lack of information.

No significant difference in efficacy has been observed between continuous infusion and bolus infusion of standard half-life recombinant FVIII (rFVIII) concentrates or plasma-derived concentrates before, during or after an invasive procedure in patients with severe hemophilia A. Continuous infusion may be associated with lower overall FVIII consumption, which can be advantageous in resource-limited settings. However, this does not apply to extended half-life rFVIII concentrates, which, according to their prescribing information, are intended for bolus administration only [1,17].

### 4.2. Patients with Hemophilia A with Inhibitors

Recommendation 1. Prophylaxis is preferable to episodic treatment of bleeding episodes (level of agreement 95.45%).

Recommendation 2. Prophylaxis with emicizumab is suggested rather than bypassing agents (level of agreement 90.90%).

Emicizumab may be more effective and cost-effective than bypassing agents in preventing hemorrhage (level of agreement 95.45%), and may offer a lower treatment burden for patients due to its weekly, fortnightly or every four weeks dosing schedule and its subcutaneous administration (with 100% agreement).

Recommendation 3. If immune tolerance induction is chosen, both low and high doses of FVIII concentrates may have a similar effect; however, low-dose regimens may be preferable in settings with limited access to FVIII (with 86.36% agreement).

Recommendation 4. In patients undergoing invasive procedures requiring treatment with bypassing agents, recombinant factor VIIa (rFVIIa) (eptacog alfa) or activated prothrombin complex concentrate (aPCC) is recommended (with 97.73% agreement). In those receiving prophylaxis with emicizumab, rFVIIa is preferable, and recommendations have currently been issued for reduced-dose aPCC (25 mg/kg body weight; maximum 100 mg/kg within 24 h) due to the possible thrombotic complications associated with concomitant use of emicizumab and aPCC (with 90.90% agreement).

Recommendation 5. In minor surgery in patients receiving treatment with emicizumab, it may be considered not to add a bypassing agent following comprehensive prior assessment, and the use of tranexamic acid may also be considered as an adjunct (with 90.90% agreement).

Recommendation 6. In major surgery, bypassing agents are recommended at doses that balance benefits and risks (with 100% agreement).

Recommendation 7. Patients with joint hemorrhage should be treated with eptacog alfa (level of agreement 97.73%), preferably with three doses of 90 μg/kg at 3 h intervals (with 88.64% agreement).

Both aPCC and eptacog alfa are approved, and in clinical practice, the choice of bypassing agent for the management of bleeding should be individualized after considering the three available options (with 100% agreement).

#### Discussion

Prophylaxis treatment with bypassing agents in PwH who have inhibitors is associated with a considerable treatment burden, requiring multiple intravenous infusions per week, and is linked to relatively high bleeding rates, in addition to being extremely costly (even more so than emicizumab). For this reason, the ideal standard of care for these patients is management with emicizumab, although other non-factor therapies such as concizumab, marstacimab or fitusiran could also be considered [16,26,27,28,29]. However, although emicizumab is highly effective as a prophylactic agent, it does not remove the requirement for bypassing agents in the event of bleeding episodes or surgical procedures. Therefore, the elimination of inhibitors aimed to restore the hemostatic efficacy of FVIII remains a key therapeutic goal [30].

The preferred approach for eliminating inhibitors is immune tolerance induction (ITI) [30]. ITI is an expensive therapy and, given that response rates obtained with high and low doses of FVIII are similar, it is preferable—especially in countries with limited resources—to opt for low-dose regimens. One way to reduce the dose of factor VIII in this case would be concomitant use with emicizumab, as described in the Atlanta protocol, which is currently being implemented in the pediatric population [31]. It is essential to determine predictors of ITI outcome and to identify in advance patients at high risk of ITI failure, thereby facilitating shared decision-making regarding discontinuation of ITI and the selection of alternative treatments, such as emicizumab, in these patients [17,30].

Performing surgery on patients with inhibitors can be very difficult and could endanger their lives, so it is essential that these patients are managed in specialized centers with experience and resources. The choice between rFVIIa or CCPa can only be based on the limited evidence provided by small cohort studies involving heterogeneous surgical settings, and it remains unclear whether either option is superior in efficacy. rFVIIa typically requires more frequent dosing and is generally more costly than CCPa, which may restrict its use in certain contexts [1]. The ISTH recommends either 3 doses of 90 μg/Kg of eptacog alfa administered at 3 h intervals or a single dose of 270 μg/kg (the panel of experts considers the first option preferable). Currently, the three bridge agents (eptacog alfa, eptacog beta and CCPa) are available on the market, so doctors should familiarize themselves with the data and prescription information of each one and take into account the warnings for the concomitant administration of CCPa and emicizumab due to the risk of thrombosis and thrombotic microangiopathy [17].

### 4.3. Patients with Hemophilia B Without Inhibitors

Recommendation 1. In severe or moderately severe Hemophilia B, prophylactic treatment is recommended over episodic treatment of bleeding episodes (with 97.73% agreement). Prophylaxis can also be considered in those with FIX levels between 2 and 5 IU/dL (agreement level 90.90%) as well as in those with baseline FIX levels of 5 to 15 IU/dL who present a severe hemorrhagic phenotype (with 97.73% agreement).

Recommendation 2. Prophylaxis can be done with purified plasma-derived FIX or standard or extended half-life rFIX concentrates (agreement level 100%). These latter ones involve a lower treatment burden for patients due to less frequent injections (with 100% agreement).

Recommendation 3. Marstacimab or concizumab may be considered, when available, as their subcutaneous administration once weekly or every 24 h, respectively, via a prefilled pen reduces the treatment burden for patients (with 95.45% agreement).

Recommendation 4. When using CCPa, it should be remembered that it can increase the risk of thrombosis (with 86.36% agreement).

#### Discussion

Prophylaxis versus episodic treatment significantly reduces hemorrhagic events and is considered a cost-effective strategy [1]. A non-randomized cohort study evaluated prophylaxis with rFIX versus prophylaxis with plasma FIX in severe PwH and suggested that both options could have a similar effect [32].

Among current treatment options, extended half-life FIX concentrates, such as nonacog beta pegol, albutrepenonacog alfa and eftrenonacog alfa, are associated with a reduced treatment burden, allowing dosing intervals of once weekly, every 10 days and, in some patients, up to every 14 or 21 days, depending on authorization in each country. Although there are significant differences in pharmacokinetics between standard half-life FIX CFCs and extended half-life ones, the clinical outcomes are similar [17].

Factor-free therapy for hemophilia B is in its early stages, with approval in some countries of marstacimab and concizumab, the two inhibitors of the anti-tissue factor pathway (anti-TFPI) that have completed phase 3 clinical evaluation. Mastarcimab was approved based on the results of the BASIS study [16] and provided an alternative to traditional factor replacement therapies, particularly benefiting PwH, which lacked SC application options [25].

Concizumab obtained FDA approval in 2025 for the treatment of PwH without inhibitors, based on the results of the explorer8 study [26], and subsequent approval for patients with inhibitors [13]. In this study, the annualized bleeding rate in PwH was reduced by about 79% (95% CI 0.10–0.45; *p* < 0.0001) with concizumab compared with no prophylaxis [26].

### 4.4. Patients with Hemophilia B with Inhibitors

Recommendation 1. PwH who develop inhibitors should receive prophylaxis with appropriate factor-free therapies when available (agreement level 93.18%).

Recommendation 2. In PwH, ITI can be considered with caution, analyzing each case individually (with 90.90% agreement).

#### Discussion

The development of high inhibitor titers against FIX complicates the treatment of bleeding episodes in PwH. For these patients, there were no easy-to-administer subcutaneous therapeutic options. Currently, the drugs marstacimab and concizumab are available, which were approved based on the results of the study BASIS [16] and explorer7 [13], respectively.

PwH often have allergic reactions, including anaphylaxis, when exposed to products containing FIX, so the experience of ITI in hemophilia B is limited, and the decision to perform it should be taken with caution [17].

## 5. Adjuvant Treatments

Recommendation 1. For the management of mucosal bleeding (oral, nasal and menstrual cavity) and surgical procedures, the use of antifibrinolytics is indicated (with 81.81% agreement).

Recommendation 2. The use of fresh frozen plasma (FFP) and cryoprecipitates should be restricted exclusively to emergency cases, when they are the only available option (with 95.45% agreement).

Recommendation 3. To control pain, discomfort or anxiety due to venous access, the use of local anesthetic spray or cream at the venous access site is recommended (with 90.90% agreement).

The World Federation of Hemophilia’s guide [4] and the consensus published in 2021 [2] specify the guidelines for pain management in PcH. According to the survey sent to the members of the panel of experts, the following considerations are made regarding management options:

Acute pain due to a joint or muscle hemorrhage:

- Paracetamol + tramadol (50%)
- COX-2 inhibitor or paracetamol + codeine (22.72%)
- Complementary measures, such as immobilization, compression and splinting, to minimize pain, where appropriate (11.36%).
- Paracetamol (9.10%)
- Strong opioid (4.54%)
- Others (2.27%)

Postoperative pain:

- Except for COX-2 inhibitors, NSAIDs are not generally recommended for use (77.27%).
- Analgesic management should follow similar principles to those applied in patients without hemophilia (22.72%).

Chronic pain due to hemophilic arthropathy in adults:

- Paracetamol + weak opioid (36.36%)
- Referral to pain management therapy (31.81%)
- Tramadol or strong opioid ± non-opioid (11.36%)
- Paracetamol (6.81%)
- COX-2 inhibitor or non-selective NSAID (6.81%)
- Education on pain management should include the use of complementary approaches for pain control, such as meditation, distraction, mindfulness or music therapy (6.81%).

### Discussion

Pain management in people with hemophilia should be based on a comprehensive and stepwise approach that combines pharmacological and non-pharmacological strategies, according to the intensity of pain, the clinical context and the presence of musculoskeletal complications [4,33].

It is recommended to initiate analgesic treatment following a stepwise regimen, beginning with non-opioid analgesics and progressing to weak or strong opioids when necessary, always accompanied by the timely administration of specific hemostatic treatment and avoiding the use of non-selective non-steroidal anti-inflammatory drugs during bleeding episodes [4,33].

Additionally, non-pharmacological interventions aimed at pain reduction and functional recovery should be promoted, including rest and temporary immobilization during acute phases, supervised physiotherapy, rehabilitation programs, muscle strengthening, application of local physical measures and patient education [4,33].

In patients with chronic pain secondary to hemophilic arthropathy, a multidisciplinary approach is recommended, integrating stepwise pharmacological treatment, physical rehabilitation, psychological support and complementary techniques, with the aim of improving functionality, reducing disability and optimizing quality of life [4,33].

In addition to coagulation factor concentrates (CFC), other agents are available that may be of considerable value in many cases. One of these is desmopressin, a synthetic analog of vasopressin that increases serum levels of FVIII and von Willebrand factor; however, its use in young children requires caution due to fluid retention. The expert group did not reach consensus regarding the situations in which its use would be recommended. Regarding antifibrinolytics, tranexamic acid and epsilon-aminocaproic acid (Amicar) are both available in Mexico [2,4,8]. These agents share a similar mechanism of action, inhibiting fibrinolysis and blocking the conversion of plasminogen to plasmin, and thereby enhancing clot stability. Amicar has a shorter plasma half-life, lower potency and a less favorable safety profile, which limits its therapeutic use. Regarding fresh frozen plasma (FFP) and cryoprecipitates, both are blood components obtained from whole blood and therefore are not subjected to viral inactivation procedures. Consequently, they carry a higher risk of transmission of viral pathogens, which becomes significant with repeated infusions; therefore, their use is restricted exclusively to emergency situations and in the absence of specific coagulation factor concentrates (CFC) [2,4].

Joint pain, whether acute due to hemarthrosis or chronic resulting from hemophilic arthropathy, represents a major clinical issue in PwH, including children and adolescents. It is estimated that up to 50% of adult PwH experience chronic joint pain that causes disability and negatively affects their quality of life. For this reason, applying appropriate pain assessment and effective management strategies is crucial in order to improve functionality and quality of life in PwH experiencing pain [4,33]. The expert group did not reach consensus on how to manage acute pain due to joint or muscle bleeding, postoperative pain and chronic pain due to hemophilic arthropathy in adults. According to World Federation of Hemophilia (WFH) recommendations, pharmacological treatment should follow a stepwise approach, avoiding non-selective NSAIDs during any phase of hemarthrosis and always accompanied by immediate factor administration and complementary measures such as immobilization [4].

The difficulty in reaching a consensus may be due to the lack of specific guidelines. In this context, the clinician makes the treatment decision based on their clinical judgment and experience. There is agreement, however, that we should rely on pain management because the goal is to control pain without increasing bleeding.

## 6. Gene Therapy

### 6.1. Eligibility

Recommendation 1. Gene therapy is indicated for individuals with severe hemophilia A (FVIII < 1 IU/dL) (with 95.45% agreement) and severe or moderately severe hemophilia B (FIX ≤ 2 IU/dL) (with 93.18% agreement), provided there is no current or previous history of inhibitors requiring ITI (with 93.18% agreement).

Recommendation 2. Eligible individuals must be able to travel to the reference center for infusion (level of agreement 95.45%) and have a caregiver or support person available (with 93.18% agreement).

Recommendation 3. Individuals with severe liver failure (uncontrolled acute or chronic hepatitis, advanced fibrosis or cirrhosis) (with 97.73% agreement), active infection (acute or chronic) (with 95.45% agreement), those who have received immunosuppressive therapy within the previous 30 days (with 90.90% agreement), and those with active inhibitors (level of agreement 86.36%) or antibodies against adeno-associated viral (AAV) vectors (level of agreement 86.36%) are not eligible candidates.

Recommendation 4. Prior to administration of gene therapy, systematic determination of anti-AAV neutralizing antibodies using validated platforms (e.g., NAb50) is recommended as part of the eligibility criteria, since pre-existing immunity to adeno-associated vectors may limit treatment efficacy and safety.

#### Discussion

The presence of neutralizing antibodies against adeno-associated viral vectors is one of the main limiting factors for eligibility for gene therapy. Several studies have shown that high titers of anti-AAV antibodies are associated with lower hepatic transduction and a significant reduction in transgene expression [34,35]. Therefore, systematic determination of neutralizing antibodies using validated platforms such as NAb50 is recommended as an integral part of pre-treatment evaluation.

### 6.2. Monitoring

Recommendation 1. The assessments that should be performed in a patient who is a candidate for gene therapy include: psychological evaluation (with 95.45% agreement) and musculoskeletal system evaluation (with 84.09% agreement), quality-of-life measures (with 90.90% agreement), baseline blood tests (with 86.36% agreement), complete blood count (with 100% agreement), and baseline liver ultrasound and FibroScan (within the previous 6 months) (with 84.09% agreement).

Recommendation 2. The patient should be monitored for at least 3 h after the infusion (with 97.73% agreement).

Recommendation 3. Between months 1–3, the patient should be monitored twice weekly (complete blood count, renal and hepatic function tests, AST, CK and FVIII/FIX) (with 97.73% agreement), and from the fourth month onwards, monitoring may be reduced to monthly, unless previous liver function tests have been abnormal (with 100% agreement).

Recommendation 4. From year 2 onwards, liver function tests should be performed every 6 months (with 100% agreement), and a liver ultrasound at 12 and 24 months (with 100% agreement).

Recommendation 5. Prior to discharge, patients should be provided with a treatment card including details of the administered therapy, contact information in case of delayed reactions, and instructions for the initial follow-up blood tests (with 97.73% agreement).

Recommendation 6. Prophylactic treatment with recombinant factor is generally continued until endogenous coagulation factor levels rise to approximately > 5 IU/dL (with 88.63% agreement).

Recommendation 7. Patients should be prescribed oral prednisolone for use in accordance with the treatment protocol (with 88.63% agreement).

Recommendation 8.l Long-term clinical, biochemical and imaging follow-up is recommended, ideally beyond 3 years after infusion, with particular emphasis on monitoring liver function tests due to reports of delayed elevations in transaminases in gene therapy clinical studies.

#### Discussion

AAV gene therapy is now an established therapeutic option for severe hemophilia A, although its clinical use remains limited [36,37]. Currently, valoctocogene roxaparvovec is the only therapy for hemophilia A approved by both the FDA and the EMA, supported by results from the GENEr8-1 study [34]. For severe and moderately severe hemophilia B, two gene therapies have received FDA approval: etranacogene dezaparvovec and fidanacogene elaparvovec, based on HOPE-B [35] and BENEGENE-2 [38] studies, respectively. In the latter trial, among 44 participants who completed at least 15 months of follow-up after receiving fidanacogene elaparvovec, the ABR for all bleeding episodes decreased by 71%, from 4.42 (95% CI: 1.80–7.05) at baseline to 1.28 (95% CI: 0.57–1.98) after gene therapy, corresponding to a difference treatment of −3.15 episodes (95% CI: −5.46 to −0.83; *p* = 0.008). These findings demonstrate both non-inferiority and superiority of fidanacogene elaparvovec compared with prophylaxis. At 15 months, mean factor IX activity was 26.9% (median: 22.9%; range: 1.9–119.0) as measured by the one-stage SynthASil assay [38].

Due to the lack of long-term follow-up, many questions remain regarding the application of gene therapy [37,39].

Experience accumulated in gene therapy clinical studies has highlighted the possibility of delayed elevations in transaminases and immune-mediated hepatic responses even several months or years after infusion. In the GENEr8-1 and HOPE-B studies, delayed-onset hepatic biochemical abnormalities have been described, which underscores the need for prolonged follow-up [34,35].

In this context, it is recommended to maintain close clinical and biochemical monitoring beyond 3 years after treatment, including periodic liver function tests and hepatic imaging studies when available, with the aim of detecting late adverse events early and preserving the long-term safety of these therapies [39].

Successful and safe treatment of people with hemophilia (PwH) with gene therapy requires a multidisciplinary approach, including education and training of the treating institutions, the establishment of patient eligibility criteria, authorization and support from insurers, acquisition and administration of the therapy, and patient monitoring and follow-up [37,39]. Given the analytical variability between laboratories in anti-AAV antibody testing, the establishment of national standardization would be beneficial.

There are numerous barriers to access to gene therapy, including age, pre-existing immunity to AAV, factor-specific inhibitors, cost, and the immediate availability of a team trained in the management of possible infusion reactions, as well as specific follow-up to ensure that immune-mediated responses do not result in loss of transgene expression [37].

### 6.3. Major Obstacles for Optimal Hemophilia Treatment in Mexico

Mexico faces multiple challenges that could potentially compromise its adherence to the international standards recommended by the World Federation of Hemophilia (WFH), despite the therapeutic advancements in hemophilia, including extended prophylaxis with long-lasting clotting factors, Emicizumab, and emerging gene therapies (Table 2) [4,40,41,42,43]. Overcoming these obstacles necessitates consistent investment, reinforcement of comprehensive healthcare centers, and increased access to care and new therapies.

## 7. Conclusions

PwH require comprehensive care that addresses all the medical and psychological aspects affecting both patients and their families. In low- and middle-income countries, there are many gaps that prevent timely access to the treatment and follow-up regimens recommended by the World Federation of Hemophilia (WFH). These include the lack of infrastructure and multidisciplinary specialist teams that facilitate timely diagnosis and appropriate treatment, as well as the high cost of therapies and limited public awareness of bleeding disorders [4,5,38]. Undoubtedly, the development of a national referral network for complex ITI, inhibitor management and access to gene therapy, as well as the inclusion of efanesoctocog alfa and anti-TFPI therapies (concizumab, marstacimab) in the institutional essential medicines list, would represent significant advances.

Mexico is currently experiencing a transition in the treatment of hemophilia due to improvements in the government procurement process for coagulation factor concentrates (CFC) and the standardization of treatment across all institutions through the Technical Protocol for the Care of Hemophilia, published in 2022. With the nationwide implementation of this protocol, supported by the health authorities, Mexico is expected to improve the care of people with hemophilia in the medium term, with a significant improvement in international indicators [44].

## Figures and Tables

**Table 1:** Approved agents for the treatment of hemophilia.

Type of Agent	Hemophilia A	Hemophilia B
Coagulation factor concentrates (CFC)	X	X
Extended half-life CFC		
*Recombinant Fc fusion proteins*		
Efmoroctocog alfa	X	
Efanesoctocog alfa	X	
Eftrenonacog alfa		X
*Recombinant albumin fusion protein*		
Albutrepenonacog alfa		X
*Pegylated rFVIII*		
Octocog alfa	X	
Turoctocog alfa pegol	X	
Damoctocog alfa pegol	X	
*Pegylated rFIX*		
Nonacog beta pegol		X
Bypassing agents (recombinant activated coagulation factor VII):		
Eptacog alfa	X	X
Eptacog beta	X	X
Non-factor therapies		
*Humanized bispecific monoclonal antibody mimicking FVIII*		
Emicizumab	X	
*Tissue factor pathway inhibitors*		
Concizumab	X	X
Marstacimab	X	X
*Antithrombin inhibitor*		
Fitusiran	X	X
Gene therapy		
Valoctocogene roxaparvovec	X	
Etranacogene dezaparvovec		X
Fidanacogene elaparvovec		X

Italics indicate the differentiate groups of drugs within each category.

**Table 2:** Barriers for optimal hemophilia treatment in Mexico.

Delayed diagnosis and underreporting	- Tendency to underdiagnose patients with mild and moderate hemophilia. - Persistent challenge of delayed diagnosis in rural areas and impoverished communities. - Lack of timely access to specialized coagulation studies. - Underreporting of patients on a nationwide scale.
Geographic disparities in healthcare access	- Concentration of specialized healthcare centers in major metropolitan areas. - Limited supply of hematologists with expertise in the treatment of hemophilia. - Patients residing in remote areas encounter significant challenges in traveling to specialized healthcare centers.
Budget constraints and unequal access to advanced therapies	- Inconsistent availability of clotting factor concentrates (CFC) among institutions. - Budget constraints regarding universal care. - Limited access to innovative therapies such as Emicizumab in some health systems. - Important differences between public and private healthcare institutions.
Medicines supply issues	- Occasional interruptions in the supply of CFCs. - Complex purchase processes. - Dependence on centralized government purchases
Development and management of inhibitors	- Limited access to inhibitor screening tests. - Limited availability of bypass agents. - High costs associated with inducing immune tolerance.
Insufficient multidisciplinary infrastructure	- Lack of comprehensive hemophilia clinics. - Limited access to specialized physical therapy. - Variable availability of orthopedics, rehabilitation, psychology, and social work services
Educational challenges	- Limited knowledge on hemophilia at primary care level. - Delays in the referral to specialized healthcare centers. - Greater need for the education of patients and caregivers.
Transition from pediatric care to adult care	- Lack of structured transition programs. - Loss of follow-up for teenagers and young adults
Home care program limitations	- Insufficient training in self-infusion for some patients. - Logistical barriers to medication distribution.
Future access to gene therapy	- Estimated high costs. - Need for highly specialized healthcare centers. - Regulatory and extended follow-up requirements.

## Data Availability

No new data were created or analyzed in this study. Data sharing is not applicable to this article.
